# Slow-Paced Breathing and Autonomic Function in People Post-stroke

**DOI:** 10.3389/fphys.2020.573325

**Published:** 2020-10-30

**Authors:** Mia Larson, Daniel P. Chantigian, Ninitha Asirvatham-Jeyaraj, Ann Van de Winckel, Manda L. Keller-Ross

**Affiliations:** ^1^Division of Rehabilitation Science, Department of Rehabilitation Medicine, Medical School, University of Minnesota, Minneapolis, MN, United States; ^2^Indian Institute of Science, Bangalore, India; ^3^Division of Physical Therapy, Department of Rehabilitation Medicine, Medical School, University of Minnesota, Minneapolis, MN, United States

**Keywords:** stroke, baroreflex, heart rate variability, slow breathing, autonomic function

## Abstract

**Purpose**: To determine if acute slow breathing at 6 breaths/min would improve baroreflex sensitivity (BRS) and heart rate variability (HRV), and lower blood pressure (BP) in adults after stroke.

**Methods**: Twelve individuals completed two randomized study visits where they performed a 15-min bout of breathing exercises at 6 breaths/min (slow) and at 12 breaths/min (control). Continuous BP and heart rate (HR) were measured throughout, and BRS, BRS response to elevations in blood pressure (BRSup), BRS response to depressions in blood pressure (BRSdown), and HRV were calculated and analyzed before (pre), during, and after (post) breathing exercises.

**Results**: BRS increased from pre to post slow breathing by 10% (*p* = 0.012), whereas BRSup increased from pre to during slow breathing by 30% (*p* = 0.04). BRSdown increased from pre to post breathing for both breathing conditions (*p* < 0.05). HR (control: *Δ* − 4 ± 4; slow: *Δ* − 3 ± 4 beats/min, time, *p* < 0.01) and systolic BP (control: *Δ* − 0.5 ± 5; slow: *Δ* − 6.3 ± 8 mmHg, time, *p* < 0.01) decreased after both breathing conditions. Total power, low frequency power, and standard deviation of normal inter-beat intervals (SDNN) increased during the 6-breaths/min condition (condition × time, *p* < 0.001), whereas high frequency increased during both breathing conditions (time effect, *p* = 0.009).

**Conclusions**: This study demonstrated that in people post-stroke, slow breathing may increase BRS, particularly BRSup, more than a typical breathing space; however, paced breathing at either a slow or typical breathing rate appears to be beneficial for acutely decreasing systolic BP and HR and increasing HRV.

## Introduction

More than 7 million Americans, equating to 2.8% of the population in the United States, suffer from a stroke during their lifetime (Benjamin et al., 2018). Autonomic dysfunction is common in people with stroke demonstrated by an attenuated heart rate variability (HRV) and impaired baroreflex sensitivity leading in part to decreased vagal modulation and increased sympathetic modulation (Nayani et al., 2016). Autonomic dysfunction increases risk for infarct expansion, cardiovascular complications, and poorer prognosis at 1 year post-stroke (Nayani et al., 2016). Thus, autonomic dysfunction is a major contributor to morbidity and mortality in people with stroke (Micieli and Cavallini, 2008).

Autonomic regulation of blood pressure (BP) occurs by modulation of heart rate (HR) and therefore cardiac output, *via* both parasympathetic and sympathetic activity and alterations to vascular reactivity *via* sympathetic activity. Cardiac baroreflex is important for buffering acute alterations in BP by increasing and decreasing cardiac output so that blood flow to organs remains relatively constant, limiting BP variability (Zhang et al., 2019). Decreased baroreflex sensitivity (BRS) after stroke corresponds to poor outcomes, independent from stroke severity at onset, stroke type, age, and resting BP (Robinson et al., 2003). Furthermore, reduced cardiac BRS increases the likelihood of developing arrhythmias, which occur in approximately 19% of patients post-stroke, 4% of which are fatal (Billman et al., 1982; Soros and Hachinski, 2012).

Therapeutic interventions targeting autonomic dysfunction, including sympathetic over-activity and blunted arterial BRS, have the potential to mitigate future risk of stroke and other co-morbidities. Current treatment strategies for baroreflex function, such as the use of β-blockers (Savitz et al., 2000; Inaba et al., 2008), are associated with the development of hyperlipidemia and insulin resistance (Carella et al., 2010) and may contribute to increased mortality and disability after stroke (Sykora et al., 2009b).

Device-guided slow breathing, in which breathing is slowed to 5–6 breaths/min, is currently a Federal Drug Administration-approved treatment for relaxation and considered an adjunctive treatment for hypertension by the American Heart Association (Brook et al., 2013; van Hateren et al., 2014). Not only is slow breathing exercises currently used as a non-pharmacologic method to reduce BP for individuals with hypertension (reviewed in Elliott and Izzo, 2006) but it has also been shown to increase cardiac BRS and HRV in healthy adults and adults with chronic heart failure (Bernardi et al., 2002; Lehrer et al., 2003). Therefore, increasing BRS and HRV may be a novel and effective target for therapy to reduce future risk of a subsequent stroke and other co-morbidities in people post-stroke (Sykora et al., 2009b). Whether slow breathing can acutely change BRS and HRV in people post-stroke, however, is not known. Slow breathing could provide a tailored rehabilitative strategy to improve BRS and HRV and therefore autonomic function in these patients. Thus, the purpose of this study was to determine if slow breathing would acutely increase BRS and HRV and decrease BP and HR in adults with chronic stroke.

## Materials and Methods

### Participants

Adults with stroke were screened if they were between ages 18 and 99, greater than 6 months post-stroke, medically stable, and had left- or right-sided hemiplegia or hemiparesis due to an ischemic or hemorrhagic stroke of the cerebrum or brainstem. Participants were excluded if they had (1) a cognitive impairment (Mini-Mental State Examination – brief version <13/16; Folstein et al., 1975), (2) a history of cardiac electrical abnormalities including atrial fibrillation, a pacemaker, or frequent premature atrial or ventricular contractions that would prevent assessment of HRV, or (3) untreated anxiety or depressive symptoms. Participants self-identified to participate in the study after seeing a flier or announcement on a university website. Participants were also recruited through local hospitals in Minneapolis, MN. The study was approved by the University of Minnesota’s Clinical Translational Science Institute and Institutional Review Board (IRB# STUDY00000821) and the procedures were performed in accordance with the Declaration of Helsinki. All participants signed a written informed consent prior to performing any study procedures.

### Experimental Design

As part of a larger study (NCT03328468), participants completed two experimental sessions at least 2 weeks apart. The experimental sessions were randomized between the slow breathing condition (6 breaths/min) and the control breathing condition (12 breaths/min). Breathing interventions were 15 min in length and completed with the assistance of a breathing application, Breathe Deep (Mineev, Bizi Apps LLC), viewed on an iPad (Apple, Cupertino, CA, USA). Breathing rates were chosen based on previous work suggesting that only rates below 10 breaths/min modulate BRS, with 6 breaths/min being optimal (Bernardi et al., 2002). For both experimental conditions, the participants followed a 2:3 inhalation-exhalation ratio and were prompted by a visual cue of the word “inhale” and a circle that enlarges with inhalation and a word “exhale” with the circle reducing in size during exhalation. Participants were continuously monitored visually by a member of the study team to ensure compliance with the breathing protocol throughout the exercise.

The participants were placed in a seated position for each visit. Participants rested for 10 min to measure baseline HR, BP, BRS, and HRV at their intrinsic breathing rate. Because slow breathing can influence ventilatory parameters and subsequently hemodynamics (Joseph et al., 2005; Dick et al., 2014), we then measured the partial pressure of end-tidal carbon dioxide, respiratory rate, ventilation, and tidal volume for 5 min through a fitted mouthpiece, nose clip, and a pre-vent pneumotach immediately before each breathing exercise (MedGraphic, St Paul, MN, USA). The participants then breathed for 15 min using the Breathe Deep application. Gas exchange and ventilation were then measured for 5 min followed by a 10-min post-breathing intervention measurement at their intrinsic rate.

HR was measured with a three-lead electrocardiography (ECG; ADInstruments, Colorado Springs, CO, USA). HR and HRV were analyzed from the ECG in 5-min increments. Blood pressure was also measured and averaged over 5-min increments with a continuous non-invasive (NIBP) monitor (ccNexfin, Edward Life Sciences, Irving, CA *or* ADInstruments, Colorado Springs, CO, USA). There was no difference between the first and second 5-min increments for all measures, and therefore, the second 5 min of the 10-min rest period and the first 5 min of the 10-min post-breathing period were used for analysis. We acquired manual BPs in the unaffected arm to perform manual corrections of BP measured by the NIBP.

### Data Analysis

Spontaneous cardiac BRS was measured using the sequence technique (Parati et al., 1988). The sequence technique identifies sequences of three or more beats in which the R–R interval and systolic BP concomitantly increase or decrease by at least 4 ms or 1 mmHg, respectively (Parati et al., 2000). Sequences of three or more beats changing in the same direction were included in the calculation if the correlation coefficient was greater than 0.7 (*r* > 0.7; Smyth et al., 1969; Rudas et al., 1999). BRS was measured over a 10-min resting period, both before and after the breathing exercises and for the duration of the 15-min breathing exercise. BRS is an average slope for both the increases in BP that correspond with increases in R–R interval (RRI) identified as up sequences (BRSup) and the reverse, decreases in BP corresponding to decreases in RRI identified, as down sequences (BRSdown). BRSup and BRSdown have been reported to correlate with the phenylephrine pressor (hypertensive stimuli) and nitroprusside depressor baroreflex test sensitivities, respectively (Watkins et al., 1995; Rudas et al., 1999).

All HRV variables were analyzed using the HRV module with LabChart software (ADInstruments, Colorado Springs, CO, USA). HRV can be measured in the time and frequency domain. Time domain HRV variables were measured as the average RRI, standard deviation of normal inter-beat intervals (SDNN), and the root mean square of successive R−R intervals (RMSSD). Power spectral analysis was used to determine the frequency domain of HRV. Frequency domain variables included total spectral power, which is a sum of high, low, and very low frequency power (0.0033–0.4 Hz). Very low frequency power is not useful for short-term variability measurements Malik et al. (1996), and its origins and mechanisms remain unclear (Berntson et al., 1997). Thus, this measure was not included in the analysis. High frequency components are in the range of 0.15–0.4 Hz and are predominantly modulated by parasympathetic activity, and low frequency components are in the range of 0.04–0.15 Hz and are under the influence of both parasympathetic and sympathetic activities (Pomeranz et al., 1985).

### Statistical Analysis

We based our *a priori* sample size calculation on baroreflex sensitivity data from a similar study in adults with hypertension (Joseph et al., 2005). Based on their means and standard deviations, slow breathing increased BRS in hypertensives from 5.8 ± 0.7 to 10.3 ± 2.0 ms/mmHg; *p* < 0.01. A large effect size (*d* = 2.56) was obtained and thus, for such a result, only a *n* = 4 would be needed to have sufficient power of 80%. Because we are investigating adults with stroke rather than people with hypertension, we estimated that the effect size could be smaller, and therefore, we conservatively chose an effect size of *d* = 0.25 to estimate the sample size for the current study (*n* = 12).

All BRS and HRV measures were log-transformed to increase normality of the data. A two-way repeated measures (RM) analysis of variance (ANOVA) was used for all outcome variables to compare the breathing conditions (slow vs. control breathing, condition effect) across time (pre, during, and post breathing, time effect). Pairwise comparisons were evaluated using the Bonferroni *post-hoc* adjustment. Prescribed medications reported by participants were separated into BP medications, anti-coagulation therapy, cholesterol medications, and muscle relaxants and were co-varied for in the statistical analysis to determine if they influenced BRS, BP, or HRV analysis. Statistical analysis was completed using SPSS (IBM SPSS Statistics version 25, SPSS, New York, NY, USA).

## Results

### Participants

**Figure 1:**
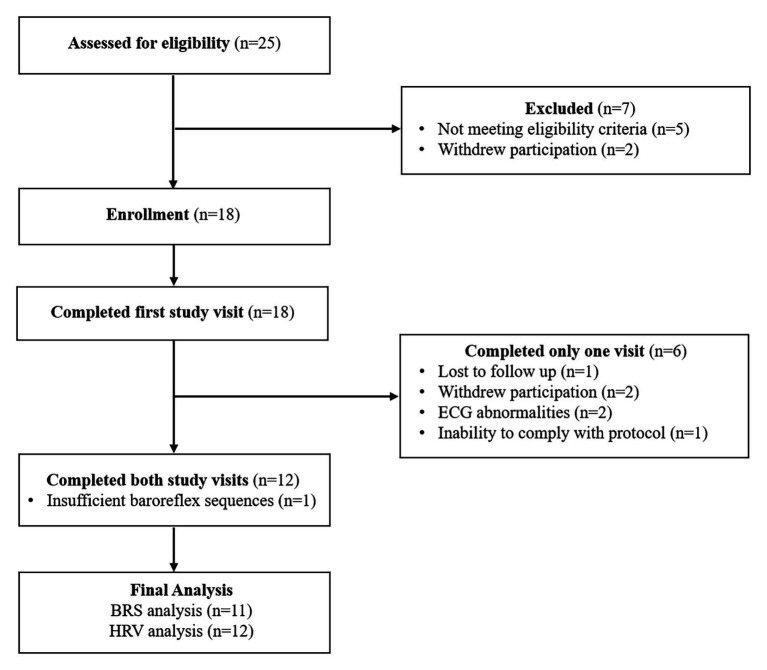
Consortium diagram. Participant disposition for the study protocol showing reasons for exclusion and ineligibility. ECG abnormalities included premature atrial and ventricular contractions occurring during greater than 3% of the total heart beats in the measurement period. The participant who was unable to comply with the study protocol was not able to slow down their respiratory rate to 6 breaths/min.

Twenty-five individuals were assessed for eligibility and 18 were recruited for the study. Twelve participants (three females, nine males; 52 ± 13 years; 91% white; average time post-stroke 3 ± 2 years) completed both study visits and were included in the final analysis (Figure 1). One of the females was premenopausal, and her menstrual cycle was not controlled for in this study. Participant characteristics and medications are listed in Table 1. One individual had insufficient baroreflex sequences and was therefore not included in the BRS analysis (BRS analysis, *n* = 11). Another individual was not able to use the pre-vent pneumotach because of facial weakness and, therefore, was excluded from the ventilatory analysis (ventilatory data analysis, *n* = 11). There were no baseline differences between the breathing conditions for any of the variables measured (Table 2).

**Table 1:** Participants’ baseline demographics, clinical characteristics, and medication use.

**Baseline characteristics**	
Number of participants (*n*)	12
Sex (men; women; *n*, percentage)	9 (75%); 3 (25%)
Race – White (*n*, percentage); Asian (*n*, percentage)	11 (91.67%); 1 (8.33%)
Ethnicity – Non-Hispanic (*n*, percentage)	12 (100%)
Age (mean ± SD)	51 ± 14.62
Ischemic stroke (*n*, percentage)	9 (75%)
Right side lesion (*n*, percentage)	11 (91.67%)
National Institute of Health Stroke Scale (mean ± SD)	2.5 ± 1.68
Time since stroke in years (mean ± SD)	3.59 ± 2.26
**Medication use**	
Statin (*n*, percentage)	8 (67.7%)
β-blocker (*n*, percentage)	2 (16.7%)
Angiotensin receptor blocker (*n*, percentage)	2 (16.7%)
Calcium channel blocker (*n*, percentage)	4 (33.3%)
Selective serotonin reuptake inhibitor (*n*, percentage)	2 (16.7%)
Baclofen (*n*, percentage)	4 (33.3%)
Angiotensin converting enzyme inhibitor (*n*, percentage)	3 (25%)
α_2_-adrenergic antagonist (*n*, percentage)	1 (8.3%)
Tricyclic antidepressants (*n*, percentage)	1 (8.3%)
Anticoagulant therapy (*n*, percentage)	6 (50%)

**Table 2:** Baseline physiological measures for the control and slow breathing conditions.

Baseline characteristics	Control breathing visit	Slow breathing visit
Heart rate	77 ± 12	74 ± 13
Systolic blood pressure	125 ± 8	126 ± 11
Diastolic blood pressure	80 ± 10	80 ± 12
RRI	801.83 ± 117.66	835.27 ± 155.06
SDRR	27.46 ± 18.0	29.06 ± 15.42
RMSSD	13.93 ± 10.30	17.60 ± 10.64
Total power	1,195.01 ± 1,864.28	966.68 ± 1,208.41
HF power	150.93 ± 301.33	137.47 ± 206.25
LF power	308.90 ± 357.87	228.12 ± 199.94
BRS (ms/mmHg)	5.74 ± 2.62	6.01 ± 3.92
BRSup (ms/mmHg)	4.62 ± 2.36	5.37 ± 4.23
BRSdown (ms/mmHg)	6.33 ± 2.83	6.43 ± 3.94
Respiratory rate (breaths/min)	13.93 ± 4.87	14.18 ± 3.82
Tidal volume (ml)	808.42 ± 474.97	748.22 ± 243.56
PETCO_2_ (mmHg)	35.81 ± 5.12	36.25 ± 6.67
Minute ventilation (l/Min)	9.41 ± 1.47	10.45 ± 4.61

BRS, baroreflex sensitivity; BRSdown, down baroreflex sensitivity; BRSup, up baroreflex sensitivity; HF, high frequency; HR, heart rate; HRV, heart rate variability; TP, total power; LF, low frequency; RMSSD, root mean square of successive difference between normal beats; RRI, R–R interval; SDRR, standard deviation between R–R intervals. *p* > 0.05 for all between baseline condition comparisons.

### Baroreflex Sensitivity

**Figure 2:**
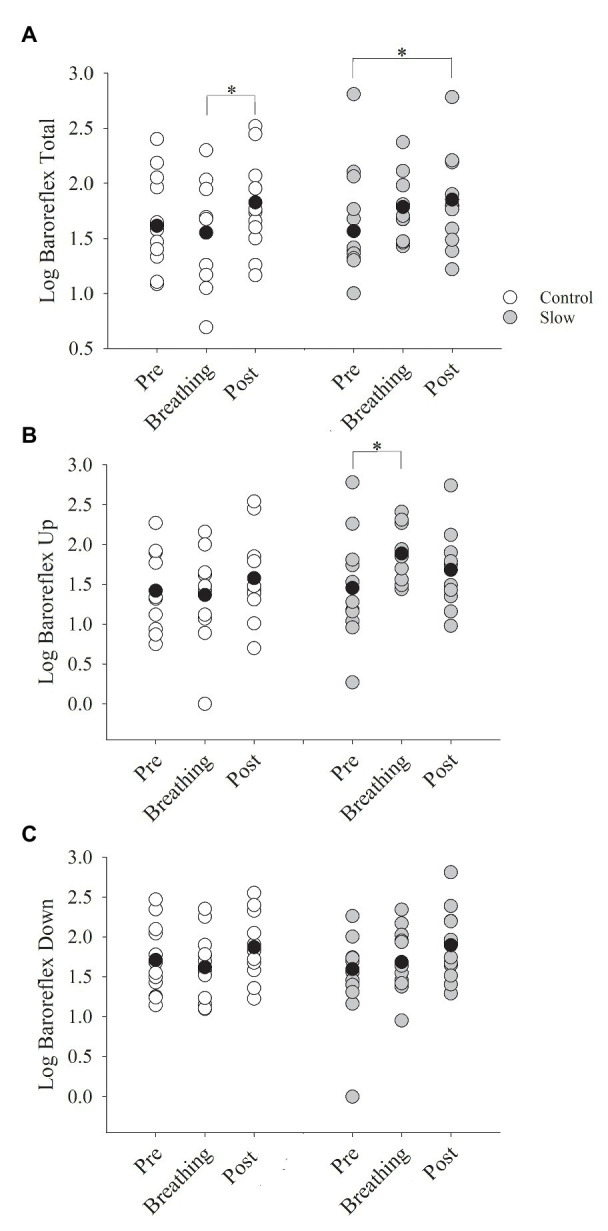
Baroreflex sensitivity (BRS) before, during, and after both breathing conditions. **(A)** BRS total increased from during breathing to post-test only for the control breathing condition (*p* = 0.01). **(B)** BRSup increased from pre-test to during breathing for the slow breathing condition only (*p* < 0.01). **(C)** BRSdown did not change for either slow or control breathing condition (*p* = 0.10). BRSup, up baroreflex sensitivity; BRSdown, down baroreflex sensitivity. Black circle indicates average data, ^*^significant difference.

Slow and control breathing differentially influenced BRS (condition × time, *p* = 0.04, Figure 2A). BRS increased in the control condition from during the breathing to post-test (time effect with Bonferroni adjustment, *p* = 0.03), likely due to the slight and non-significant decline in BRS during breathing, relative to pre-testing. BRS during the slow breathing condition increased from pre- to post-test (*p* = 0.01). BRSup was also differentially influenced by slow and control breathing (condition × time, *p* = 0.02, Figure 2B). In contrast to BRS, however, the control breathing had no effect on BRSup, but slow breathing increased BRSup from pre-breathing to during breathing (time effect with Bonferroni adjustment, *p* = 0.04) and returned to pre-breathing levels during the post-test (*p* = 0.12). BRSdown increased during both slow and control breathing conditions (time effect, *p* = 0.002) similarly (condition × time, *p* = 0.94, Figure 2C). A pairwise comparison with a Bonferroni adjustment indicated that for both conditions combined, there was an increase in BRSdown from breathing to post-test (*p* = 0.002). When co-varying for each group of medications separately in the BRS analysis, all interactions remained.

### Heart Rate, Blood Pressure, and Gas Exchange

**Figure 3:**
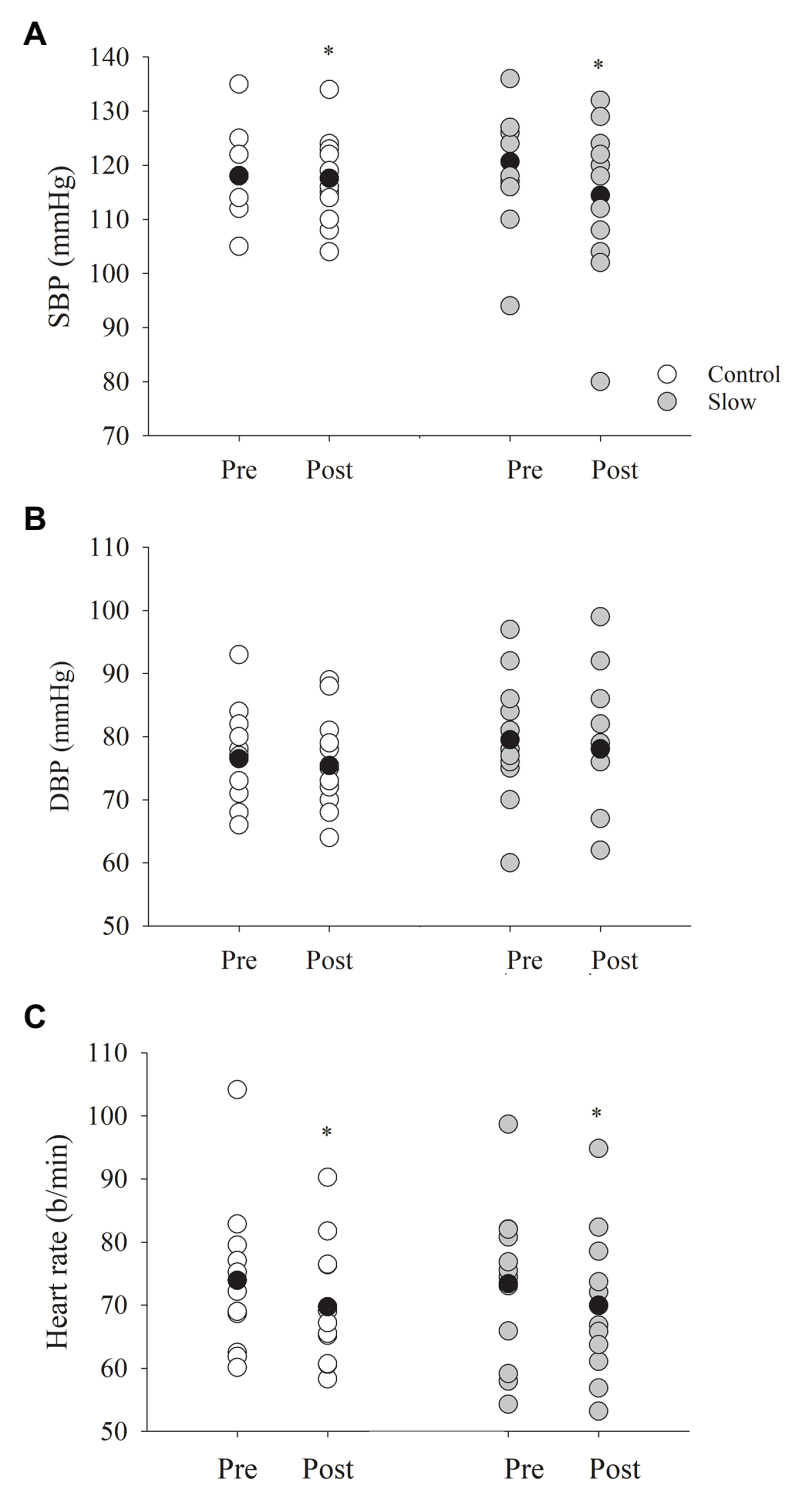
Blood pressure and heart rate (HR) before and after both breathing conditions. **(A)** Systolic blood pressure (SBP) decreased from pre- to post-test for both breathing conditions (*p* = 0.002), where diastolic blood pressure (**B**, DBP) did not (*p* = 0.44). **(C)** HR decreased following each breathing condition (*p* < 0.001). Black circles indicate average data, ^*^significant difference.

Systolic blood pressure decreased from pre- to post-breathing (time effect, *p* = 0.002, Figure 3A) similarly in both the control and slow breathing conditions (condition × time, *p* = 0.13). Diastolic blood pressure, however, did not change from pre- to post-breathing (time effect, *p* = 0.44, Figure 3B) for either breathing condition (condition × time, *p* = 0.80). Thus, mean arterial pressure was not influenced by either of the breathing conditions (control: *Δ* − 0.89 ± 4.04; slow: *Δ* − 3.03 ± 5.42 mmHg; time effect, *p* = 0.12; condition × time *p* = 0.20). There was a decrease in HR (time effect, *p* < 0.001, Figure 3C) that was similar for both the slow and control breathing conditions (condition × time, *p* = 0.37). Co-varying for each group of medications in the statistical analysis did not influence the decrease in BP or HR.

Ventilation did not change after either of the breathing conditions (control: *Δ*1.41 ± 3.78; slow: *Δ* − 0.77 ± 4.0 l/min; time effect, *p* = 0.80; condition × time, *p* = 0.13). Similarly, respiratory rate (control: *Δ*1.24 ± 2.49; slow: *Δ*0.74 ± 4.61 breaths/min; time effect, *p* = 0.31; condition × time, *p* = 0.70) and tidal volume (control: *Δ* − 29.49 ± 117.55; slow: *Δ* − 76.91 ± 208.57 ml; time effect, *p* = 0.22; condition × time, *p* = 0.46) were not influenced by either of the breathing conditions. Partial pressure of end-tidal carbon dioxide (P_ET_CO_2_) was similar before and after breathing (time effect, *p* = 0.20) in both breathing conditions (control: *Δ* − 2.77 ± 3.96; slow: *Δ*0.11 ± 4.09 mmHg, condition × time, *p* = 0.08).

### Heart Rate Variability

**Figure 4:**
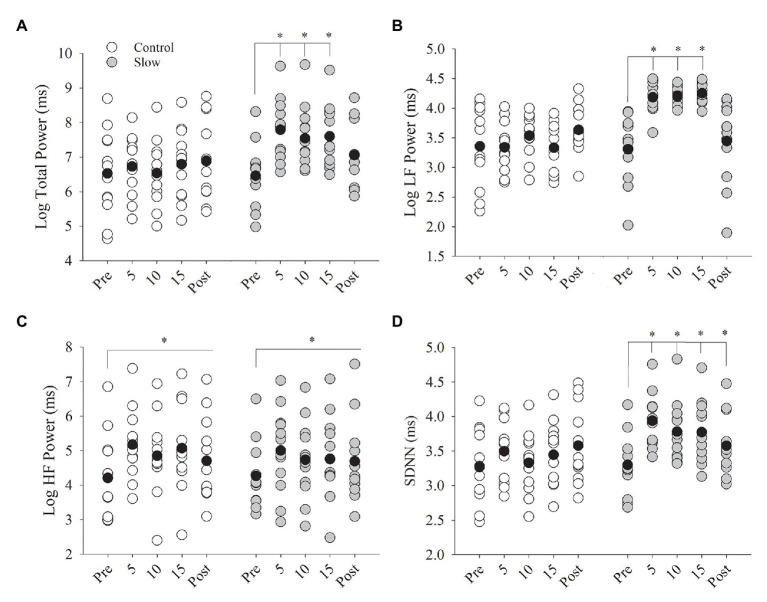
Heart rate variability (HRV) before, during, and after both breathing conditions. **(A)** Total power and **(B)** low frequency power (LFpower) increased from pre-breathing to 5, 10, and 15 min of breathing (*p* < 0.001) but returned to pre-test levels in the post-test (*p* = 0.18). **(C)** High frequency power (HFpower) increased from pre to during breathing and post-test for both control and slow breathing conditions (*p* = 0.003). **(D)** SDNN increased from pre-test to 5, 10, and 15 min of breathing (*p* < 0.05) and remained elevated during post-test (*p* < 0.05). Black circles indicate average data, ^*^significant difference.

Slow and control breathing conditions differentially influenced total power (condition × time, *p* < 0.001, Figure 4A). Total power was increased during the 15 min of slow breathing (time effect with Bonferroni adjustment for multiple comparisons, *p* < 0.001) and returned to baseline after the breathing exercise (*p* = 0.18) but did not change during the 15 min of control breathing (time effect, *p* = 0.37). Similarly, low frequency power (LF power) was differentially influenced by the breathing conditions (condition × time, *p* < 0.001, Figure 4B). LF power was increased during the 15-min slow breathing condition (time effect, *p* < 0.001) and returned to baseline after the breathing exercise (*p* = 1.0) but did not change during the control breathing condition (time effect, *p* = 0.12). High frequency power (HF power) increased during both breathing conditions (time effect, *p* = 0.003) similarly (condition × time, *p* = 0.63, Figure 4C) and remained elevated during post-testing for both breathing conditions combined (*p* = 0.02). Slow and control breathing conditions differentially influenced SDNN (condition × time, *p* = 0.001, Figure 4D). SDNN increased during the slow breathing condition only and remained elevated above baseline during post-testing (time effect with Bonferroni adjustment for multiple comparisons, *p* < 0.05). RRI increased with breathing (time effect, *p* < 0.01) similarly for both conditions from pre- to post-breathing (control: *Δ*0.05 ± 0.04; slow: *Δ*0.04 ± 0.05 ms, condition × time, *p* = 0.67). RMSSD did not change after either breathing condition (control: *Δ*3.97 ± 5.66; slow: *Δ*4.24 ± 6.62 ms; condition × time, *p* > 0.05 for all comparisons).

## Discussion

The novel findings from this study were that slow breathing acutely increases BRS, and in particular, BRSup, total power, LFpower, and SDNN. In addition, paced breathing at a slow and typical rate increases BRSdown and HFpower and decreases BP and HR. Although the BP was not differentially altered by slow vs. controlled breathing, it is important to note that the control breathing decreased systolic BP by 0.5 mmHg and the slow breathing decreased systolic BP by 6.3 mmHg, which is considered a clinically meaningful decrease in BP (Turnbull and Blood Pressure Lowering Treatment Trialists’ Collaboration, 2003; Wong and Wright, 2014). Thus, paced breathing, and in particular, slow breathing, may be beneficial for improving baroreflex function, HRV, and decreasing BP in people post-stroke.

### Evidence of Autonomic Dysfunction in People Post-stroke

It has been widely demonstrated that people post-stroke exhibit autonomic dysfunction. Indeed, Robinson et al. (2003) demonstrated that after stroke, those with an impaired BRS had a greater risk of mortality (28%) compared with controls (8%). It is also true that a reciprocal relationship may exist between stroke and autonomic dysfunction, such that autonomic dysfunction can contribute to stroke and a stroke can exaggerate autonomic dysfunction. Two leading contributors to stroke, atherosclerosis and hypertension, are both diseases accompanied by autonomic dysfunction, in which they exhibit an increase in sympathetic activity and a decrease in BRS and parasympathetic function (Yperzeele et al., 2015). Atherosclerosis is considered an inflammatory disease (Gidron et al., 2007), and the vagal nerve plays an important role in inflammation through the “cholinergic anti-inflammatory pathway” (Tracey, 2002). This suggests that altered vagal function (parasympathetic function) may contribute to greater inflammation and therefore atherosclerosis. Therefore, it is possible that poor vagal nerve function can increase the risk for stroke. As such, it has been suggested that reductions in HRV and baroreflex function often observed in individuals post-stroke may have been present prior to the stroke (Robinson et al., 1997). Thus, because autonomic dysregulation may be further impaired by having a stroke, individuals with a previous stroke are at higher risk for a subsequent stroke (Benjamin et al., 2018). The increased risk of a subsequent stroke elevates the importance and need for rehabilitative strategies to improve autonomic function after stroke.

Lateralization of stroke likely plays an important role in determining the influence or magnitude of impact the stroke has on autonomic dysfunction. The majority of literature suggests that right-sided lesions cause greater autonomic dysregulation vs. left-sided lesions (Korpelainen et al., 1996b; Zhang and Oppenheimer, 1997; Henderson et al., 2004; Ay et al., 2006; Walter et al., 2013), but this is not always a consistent finding (Sykora et al., 2009a). For example, right hemispheric infarcts have been shown to increase nocturnal blood pressure, reduce circadian BP variability, and is associated with both a higher norepinephrine level and reduced HRV, compared with left hemisphere infarcts (Korpelainen et al., 1996b; Ay et al., 2006). As such, HRV is depressed in individuals who suffered a right-sided lesion compared with a left-sided lesion (Walter et al., 2013). Baroreflex impairment, however, has been demonstrated in left-sided insular involvement (Sykora et al., 2009a). In our study, all but one of the participants experienced a right-sided lesion, indicating a likely possibility that stroke influenced autonomic function.

### Mechanisms of Slow Breathing on Autonomic Function in Stroke

Our study suggests that BRS and BRSup increased with slow breathing and that paced breathing at both 6 and 12 breaths/min increased BRSdown. BRS is generally quantified as the averaged sensitivity of the baroreflex response to both increases and decreases in BP (La Rovere et al., 2008). BRSup corresponds to the rapidity in which cardiovagal tone responds to the increases in BP and is highly correlated with the phenylephrine pressor response, which is the baroreflex response to hypertensive stimuli (Watkins et al., 1995; Rudas et al., 1999). Alternatively, BRSdown corresponds to a reduction in vagal nerve activity when BP decreases and is correlated with the nitroprusside depressor sensitivity test (Watkins et al., 1995; Rudas et al., 1999). A depressed BRSup (but not BRSdown) was found to be a significant predictor of the propensity for developing fatal arrhythmias after a heart attack in dogs 3–4 weeks after a myocardial infarction (Billman et al., 1982). This suggests that an attenuated BRSup may be linked to arrhythmias and that distinguishing between BRSup and BRSdown may be of importance. To this end, there is an asymmetry of the cardiac baroreflex that suggests that it buffers arterial pressure increases more efficiently than arterial pressure decreases (De Maria et al., 2019). This has been shown in a greater alteration in viscoelastic properties of the barosensory vessels, leading to a larger carotid artery diameter and stretch of these vessels upon the increase of systolic arterial pressure (Bonyhay et al., 1997; O’Leary et al., 2005). As such, when we separate BRS into BRSup and BRSdown, we find that BRSup is influenced by slow breathing only, whereas BRSdown is influenced by both control and slow breathing conditions. BRS increased from during breathing to post-test for the control breathing condition, but this is likely because there was a non-significant decrease during breathing, relative to pre-breathing in the control breathing condition. Collectively, these findings suggest that the increase in BRSup with slow breathing may be a beneficial strategy to attenuate acute surges in BP in people post-stroke by causing greater arterial vasodilation.

Our findings that slow breathing can increase BRS in people post-stroke are consistent with others that have studied these effects in other chronic conditions, such as hypertension (Joseph et al., 2005; Hering et al., 2013), heart failure (Bernardi et al., 2002), and post-traumatic stress disorder (Fonkoue et al., 2018). Baroreflex is important for mitigating fluctuations in BP variability, which can contribute to end organ vessel damage and increase stroke risk independent of baseline BP (Mancia and Parati, 2003; Zhang et al., 2019). The effect of slow breathing on BRS is thought to occur *via* resonance in the phase of BP changes, respiratory rate, and HR fluctuations (Lehrer and Gevirtz, 2014). These relationships between HR, BP, and respiration are well known as cardiorespiratory coupling (Dick et al., 2014). Tidal volume during inspiratory slow breathing increases substantially to compensate for the decreased respiratory rate (Anderson et al., 2009). Consequently, during slow breathing, inspiration activates pulmonary vagal afferents that reflexively inhibits sympathetic nerve discharge (Seals et al., 1990). Taking together our findings and findings from previous studies (Goso et al., 2001; Bernardi et al., 2002; Joseph et al., 2005; Harada et al., 2014), it appears that slow breathing induces a generalized decrease in the excitatory pathways regulating respiratory and cardiovascular systems. Further, respiratory and cardiovascular systems share similar control mechanisms; thus, alterations in one system will modify the functioning of the other (Somers et al., 1991; Francis et al., 2000).

Slow breathing exercises have also been shown to reduce systolic BP in individuals with hypertension (Joseph et al., 2005; Hering et al., 2013). However, paced breathing at typical rates (15 breaths/min) also resulted in BP decreases of comparable magnitude (Joseph et al., 2005). Consistent with these studies, in the current study, both the slow and control breathing conditions reduced systolic BP as well as HR in people post-stroke, suggesting the importance of paced breathing. In contrast, however, this reduction in BP has not been shown in all studies (Altena et al., 2009). It is important to note that even though a significant interaction between how the different breathing rates influenced BP was not observed in our study, control breathing resulted in a 0.5-mmHg reduction in BP where slow breathing resulted in a 6.3-mmHg reduction in BP. Thus, although the decrease in BP was not statistically different between the two groups, the decrease in BP with slow breathing is clinically meaningful (Turnbull and Blood Pressure Lowering Treatment Trialists’ Collaboration, 2003; Wong and Wright, 2014). The decrease in BP is likely modulated by the increase in BRS and subsequent activation of parasympathetic and inhibition of sympathetic activity. In veterans with post-traumatic stress disorder, slow breathing acutely increases the sympathetic baroreflex response and decreases sympathetic activity (Fonkoue et al., 2018). Although we did not measure sympathetic activity in this study, a reduction in sympathetic activity particularly during the slow breathing condition could explain the reduction in BP that we observed. The decrease in BP after the breathing exercises are likely not due to alterations in ventilation or gas exchange as has been shown in previous studies (Anderson et al., 2009), because there were no acute changes in ventilation or P_ET_CO_2_ immediately after slow breathing.

Slow breathing increased HRV in the adults post-stroke. Total power was increased after slow breathing which was likely a consequence from an increase in both HFpower and LFpower. However, there was a significantly greater increase in LFpower with slow breathing with an increase in HFpower for both breathing conditions. The increase in LFpower that we observed in our study during slow breathing is expected due to cardiorespiratory coupling and, thus, respiratory sinus arrhythmia (RSA). Respiratory sinus arrhythmia is HRV in synchrony with the phase of respiration, in which RRI is shortened during inspiration and lengthened during expiration (Berntson et al., 1993; Yasuma and Hayano, 2004), and thus, respiration has a profound impact on HRV. During typical breathing rates as in the control breathing condition, RSA has a frequency of 0.25 Hz. With slow breathing at 6 breaths/min, RSA frequency shifts in phase from HFpower to LFpower (Angelone and Coulter, 1964). LFpower reflects both sympathetic and vagal influences related to baroreflex mechanisms. Changes in the magnitude of LFpower are likely due to variations in the vagal-mediated baroreflex response from subsequent alterations in amplitude of sympathetic blood pressure rhythms or by changes in sensitivity of baroreceptors (Berntson et al., 1997; Cevese et al., 2001). The mechanisms of cardiorespiratory coupling are several fold. There is evidence to suggest that atrial and pulmonary stretch receptors, likely from the Hering-Breuer reflex with substantial increases in lung inflation during inspiration (Taha et al., 1995), solicit more efficient respiratory center and autonomic center activity (Seals et al., 1990). This results in the action of the respiratory centers modulating activity and responsiveness of vagal motoneurons and thus HR (Eckberg, 2003). Thus, previous studies suggest that slow breathing leads to a stronger cardiorespiratory coupling, and the current findings indicate that this coupling is preserved in adults post-stroke. Although, we did not measure respiration during the paced breathing protocol, the increase in HRV with slow breathing is consistent with previous findings in healthy adults (Angelone and Coulter, 1964; Porta et al., 2018) and those with heart failure (Porta et al., 2018). HFpower represents an increase in parasympathetic activity which is modulated by vagal nerve activity (Malik and Camm, 1993; Berntson et al., 1997). Therefore, our findings suggest that slow breathing increases LFpower because of a phase shift in RSA and a subsequent change in baroreflex sensitivity and stronger cardiorespiratory coupling, and both breathing conditions (at 6 and 12 breaths/min) increased cardiovagal activity suggesting a greater responsiveness of vagal motoneurons causing an activation of parasympathetic activity. Only one other study investigated slow breathing in adults post-stroke and did not find that breathing at a rate of 6 breaths/min increased HRV compared with controls. A major difference between our study and Beer et al. (2017) is that their breathing protocol was only 2 min in duration, which is not likely long enough to observe effects that the slow breathing intervention may have induced.

### Limitations

This study has limitations to be considered when interpreting the data. Our study investigated the effects of one bout of slow breathing on autonomic function. As such, greater changes to BRS with slow breathing are observed after several weeks of practice rather than just one episode (Lehrer et al., 2003). In addition, while the effect of one 15-min bout on BP may be modest, a more profound reduction has been shown to occur with daily slow breathing over several weeks (Brook et al., 2013). We are unable to determine if our study participants exhibited autonomic dysfunction, but autonomic dysfunction in people post-stroke is well documented in the literature (Korpelainen et al., 1996a,b; Bassi et al., 2007; Brook et al., 2013; Chen et al., 2013; Nayani et al., 2016). Medications can influence outcomes that were measured in this study (Sykora et al., 2009b); however, when co-varied for medications in the statistical analysis, they did not appear to influence the results of the study and medications did not vary from control to slow breathing conditions. To elucidate direct mechanisms of the stronger cardiorespiratory coupling with slow breathing in adults post-stroke, it will be important for future studies to measure respiratory rate during paced breathing conditions (Porta et al., 2018). In final, this study was conducted in conjunction with a larger study, and therefore, the time of day and fasting conditions were not controlled.

## Conclusion

Findings from this study demonstrate that slow breathing acutely increases BRS, and in particular, BRSup. Total power, LFpower, and SDNN, also increased during slow breathing, but paced breathing at a slow and typical rate increases BRSdown and HFpower and decreases BP and HR. The greater increase in BRSup with slow breathing is particularly important, given that this could result in a greater reduction in acute BP in periods of cardiovascular stress. The decrease in BP observed in the slow breathing condition was not statistically significant from the control breathing condition but is a clinically meaningful reduction in BP. As such, slow breathing may be a beneficial technique for clinicians to implement in their care for patients to improve baroreflex function, HRV, and BP in patients after stroke. Further studies to investigate the long-term influence of slow breathing in people post-stroke are warranted and would provide direction on the use of slow breathing exercises as a potential rehabilitative treatment to improve autonomic function in people post-stroke.

## Data Availability Statement

The raw data supporting the conclusions of this article will be made available by the authors, without undue reservation.

## Ethics Statement

The studies involving human participants were reviewed and approved by University of Minnesota Institutional Review Board. The patients/participants provided their written informed consent to participate in this study.

## Author Contributions

ML, AVdW, and MK-R conceived and planned the experimental design, analyzed the data, and wrote the manuscript. DC and NA-J assisted in data collection. AVdW assisted with recruitment and coordination of scheduling. All authors contributed to the article and approved the submitted version.

### Conflict of Interest

The authors declare that the research was conducted in the absence of any commercial or financial relationships that could be construed as a potential conflict of interest.

## Abbreviations

BP: Blood pressure
BRS: Baroreflex sensitivity
BRSdown: Down baroreflex sensitivity
BRSup: Up baroreflex sensitivity
LFpower: Low frequency power
HFpower: High frequency power
HR: Heart rate
HRV: Heart rate variability
P_ET_CO_2_: Partial pressure of end-tidal carbon dioxide
RMSSD: Root mean square of successive difference between normal beats
RRI: R–R interval
RSA: Respiratory sinus arrhythmia
SBP: Systolic blood pressure
SDNN: Standard deviation between R–R intervals (with ectopy removed)
